# Haematopoietic and immune defects associated with *GATA2* mutation

**DOI:** 10.1111/bjh.13317

**Published:** 2015-02-23

**Authors:** Matthew Collin, Rachel Dickinson, Venetia Bigley

**Affiliations:** Human Dendritic Cell Laboratory, Institute of Cellular Medicine, Newcastle UniversityNewcastle upon Tyne, UK

**Keywords:** *GATA2*, bone marrow failure, immunodeficiency

## Abstract

Heterozygous familial or sporadic *GATA2* mutations cause a multifaceted disorder, encompassing susceptibility to infection, pulmonary dysfunction, autoimmunity, lymphoedema and malignancy. Although often healthy in childhood, carriers of defective *GATA2* alleles develop progressive loss of mononuclear cells (dendritic cells, monocytes, B and Natural Killer lymphocytes), elevated FLT3 ligand, and a 90% risk of clinical complications, including progression to myelodysplastic syndrome (MDS) by 60 years of age. Premature death may occur from childhood due to infection, pulmonary dysfunction, solid malignancy and MDS/acute myeloid leukaemia. *GATA2* mutations include frameshifts, amino acid substitutions, insertions and deletions scattered throughout the gene but concentrated in the region encoding the two zinc finger domains. Mutations appear to cause haplo-insufficiency, which is known to impair haematopoietic stem cell survival in animal models. Management includes genetic counselling, prevention of infection, cancer surveillance, haematopoietic monitoring and, ultimately, stem cell transplantation upon the development of MDS or another life-threatening complication.

GATA binding protein 2 (GATA2) is a key transcriptional regulator of haematopoiesis required for the development and maintenance of a healthy stem cell pool. Mutation of *GATA2* has long been predicted to be relevant to leukaemogenesis but the human syndromes of GATA2 deficiency have only been recently described. Clinical phenotypes include patients with hereditary myelodysplastic syndrome (MDS) and acute myeloid leukaemia (AML) but also protean manifestations of immunodeficiency, neoplasia, lymphoedema and extra-haematopoietic defects.

In this review we summarize the molecular biology, clinical, haematological and immunological features that arise and discuss potential strategies for clinical management.

## *GATA2* gene structure and regulation

**Figure 1:**
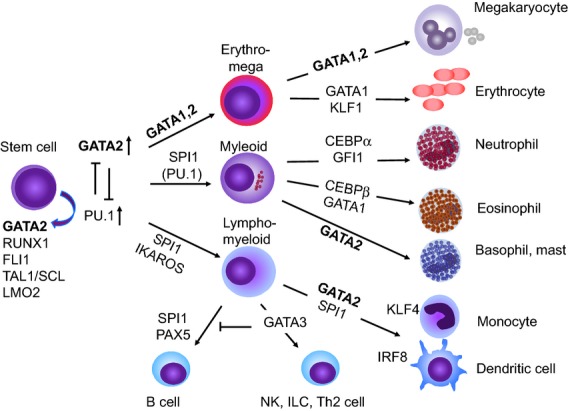
Role of GATA2 in haematopoietic differentiation. Simplified map of key interactions of GATA2 with selected major lineage-specifying transcription factors. The factors indicated are necessary for downstream differentiation according to knock-out models or are highly expressed in differentiated cells but the figure does not contain an exhaustive list of all the transcription factors that have been implicated. Antagonism between pairs of transcription factors is a notable feature of fate decisions, as exemplified by GATA2 and SPI1 (PU.1) in influencing the spectrum of early commitment. In the ‘GATA switch’ GATA2 is replaced by GATA1 at erythroid-specific sites. For more details and source information see: (Orkin, 2000; Orkin & Zon, 2008; Bresnick *et al*, 2010; Rodrigues *et al*, 2012).

GATA2 is one of six GATA binding-factors that regulate gene expression by binding to the DNA motif GATA and other transcription factors via two zinc finger domains (Orkin, 2000; Bresnick *et al*, 2010; Rodrigues *et al*, 2012). In the embryo, GATA2 is pivotal in the endothelial to haematopoietic transition that produces the first adult haematopoietic stem cells (HSCs) and consequently, homozygous knock-out is lethal due to the failure of definitive haematopoiesis (Tsai *et al*, 1994). In adult haematopoiesis, GATA2 is required for HSC survival and self-renewal, interacting with a complex network of transcription factors that specify early lineage commitment, including SPI1 (PU.1), FLI1, TAL1 (SCL), LMO2 and RUNX1, among others (Dore *et al*, 2012; Beck *et al*, 2013; May *et al*, 2013). During haematopoietic differentiation, GATA2 is likely to play a key role in downstream fate decisions together with CEBPα, GATA1 and SPI1 and is expressed in mature megakaryocytes, mast cells and monocytes (Fig1).

The *GATA2* gene is situated on the long arm of human chromosome 3 at position 21.3 and its expression is regulated at multiple levels. Enhancers at −110 kb (77 kb in mouse) and in intron 5 (intron 4 in mouse) are required for appropriate haematopoietic expression (Martowicz *et al*, 2005; Grass *et al*, 2006; Khandekar *et al*, 2007; Brandt *et al*, 2008). Multiple binding sites for GATA1 and GATA2 are found within the regulatory regions of GATA2, including the −110 kb and +9·5 kb intronic enhancers (Martowicz *et al*, 2005; Grass *et al*, 2006; Khandekar *et al*, 2007; Snow *et al*, 2011; Lim *et al*, 2012; Gao *et al*, 2013). The intronic enhancer contains an E box GATA composite element, which mediates assembly of a complex containing GATA1 or GATA2, TAL1, LIM domain binding protein (LDB1) and LIM domain only 2 (LMO2). SMARCA4 (BRG1), the ATPase component of the SWI/SNF complex, may also act with LDB1 to establish and maintain GATA2 expression by keeping the +9·5 site in open chromatin configuration (Sanalkumar *et al*, 2014). In addition, *GATA2* transcription is regulated by several loci including CEBPA, HOXA9, ETS1, BMP4, NOTCH1, SPI1 and EVI1 and by cytokines IL1 and TNFα (Vicente *et al*, 2012).

GATA2 cooperates with six other factors (TAL1, LYL1, LMO2, ERG, FLI1 and RUNXI) to form a core heptad regulatory unit bound to over 1000 loci in primitive haematopoietic cells (Wilson *et al*, 2010). Heptad target genes include microRNAs and are lineage and maturation stage-specific. Direct targets of GATA2 itself include, GATA1, SPI1 and CEBPA, together with the heptad factors TAL1, LMO2, FLI1 and RUNX1. The GATA2 protein interacts directly with ZFPM1 (FOG1), SPI1 and CEBPA (Vicente *et al*, 2012).

Three *GATA2* transcripts have been described. Expression of the distal first exon, IS, is haematopoietic-restricted and involved in specification of definitive HSCs during embryogenesis (Minegishi *et al*, 1999; Pan *et al*, 2000; Kobayashi-Osaki *et al*, 2005). Two protein isoforms have been described; isoform 1 with 480 residues and a shorter isoform 2 which is truncated by 14 residues at the second zinc finger, due to alternative splicing of the last exon. GATA2 may be modified by phosphorylation, acetylation and sumoylation and is rapidly turned over by ubiquitination (Towatari *et al*, 1995; Hayakawa *et al*, 2004; Minegishi *et al*, 2005).

## Haplo-insufficiency of GATA2

The level of expression of GATA2 relative to other transcription factors is important in gene regulation and cell fate decisions. GATA factors can bind to DNA as monomers or dimers and their configuration is likely to be concentration-dependent (Bates *et al*, 2008). Inactivation of one *GATA2* allele, or haplo-insufficiency, induces defects of haematopoiesis in animal models. The production of mouse HSCs and performance of HSCs in serial or competitive transplantation assays is inferior and there is perturbation of the granulocyte-macrophage colony-forming unit compartment (Ling *et al*, 2004; Rodrigues *et al*, 2005, 2008). Insufficient GATA2 appears to allow HSCs to enter cell cycle and differentiate, thereby depleting self-renewal capacity (Ezoe *et al*, 2002; de Pater *et al*, 2013), while over-expression impairs haematopoiesis by blocking differentiation (Heyworth *et al*, 1999; Persons *et al*, 1999; Tipping *et al*, 2009). Fine-tuning of the balance between self-renewal and differentiation of HSC appears to be a critical function of GATA2, possibly through its role in mediating contact-dependent quiescence signals from the BM niche (de Pooter *et al*, 2006; Guiu *et al*, 2013). Direct effects on apoptosis are also thought to be mediated by an interaction of GATA2 with BCL2L1 (BCL-XL) (Rodrigues *et al*, 2005). The consequences of *GATA2* haplo-insufficiency upon HSC equilibrium are more strikingly revealed in humans than mice, owing to the greater longevity of haematopoiesis.

## Heterozygous mutation of *GATA2* in humans

**Figure 2:**
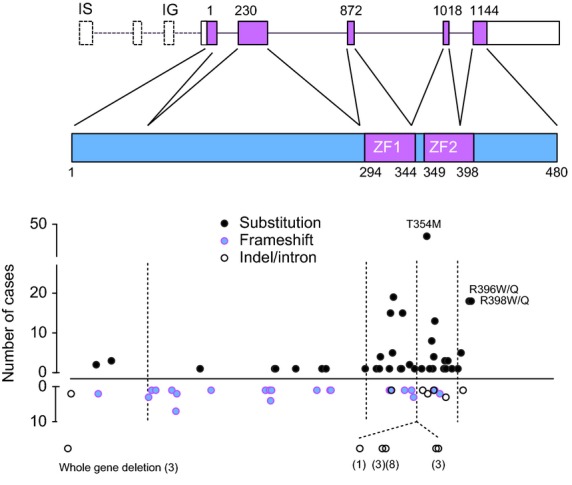
Gene structure, transcripts and frequency of mutations detected in *GATA2*. See Table SI for full details. Three *GATA2* transcripts have been described in humans: NM_032638.4 (3383 bp) and NM_001145662.1 (3263 bp) both contain six exons using alternative first exons IS and IG, respectively. A third transcript NM_001145661.1 (3484 bp) uses an intervening exon and IG (seven exons) but encodes the same isoform 1 as NM_032638.4 (480 amino acids). NM_001145662.1 is translated into a shorter isoform 2 (466 residues). Transcription of exon IS is haematopoietic specific. Mutations in the fifth intron either affect splicing and cause deletions (1017 + 2 and 1018 − 1) or are localized to the enhancer region (1017 + 512 and 1017 + 572). Annotation of the gene indicates the first base of each exon, numbered from the transcript; annotation of the protein indicates the first and last residue of the zinc fingers (ZF); broken lines show the position of exon boundaries relative to the amino acid sequence. Numbers in parentheses refer to the number of cases with intron mutations.

Nearly 100 *GATA2* mutations have been described, either as germ-line genetic defects or somatic mutations in association with other drivers, such as biallelic *CEBPA* mutation in AML (Fig2, Tables I and SI). Approximately one-third of all germ-line mutations are inherited and the rest occur *de novo*. These include a small number of whole gene deletions and 29 frame-shift or nonsense mutations, distributed from the initiation site to the end of the second zinc finger. A further 11 in-frame insertions or deletions and 54 single nucleotide variants causing amino acid substitution are concentrated in exons 3, 4 and 5, encoding the two zinc finger domains. Splice site mutations are also found between coding exons 3 and 4. Two discrete mutations of the intron 5 enhancer, predicted to affect transcription factor binding, have also been reported. Overall, approximately two-thirds of all cases described have mutations in the zinc finger domains (Fig2). No mutations have been observed in the 5′ or 3′ untranslated regions (UTRs) or in the distal section of the last exon, beyond the region encoding the second zinc finger. In order to capture all of the reported single base changes, small insertions and deletions, it is necessary to sequence codons 1–398, together with the intron 5 enhancer.

**Table I:** Mechanisms of GATA2 deficiency

Type of defect (location)	Reported mechanisms	Pobable effect
Gene deletion	Whole gene deletion	Haplo-insufficiency due to hemizygosity with absent transcription of one allele*
Regulatory mutation (non-coding regions)	Mutation *FLI1* site in the intron 5 enhancer inv 3 or t(3;3) resulting in translocation of the *GATA2* distal haematopoietic enhancer	Haplo-insufficiency due to reduced transcription of one allele*
Frameshift mutation (across coding region)	Nonsense-mediated decay† Premature stop codon Disrupted splice site‡	Haplo-insufficiency due to loss of expression or severe truncation of GATA2 protein*,‡
Single nucleotide polymorphism (concentrated in zinc finger regions)	Non-functional or hypo-functional protein (unable to bind DNA or transactivation partners) Dominant negative functional protein	Haplo-insufficiency due to expression of GATA2 protein with reduced function*,‡

* Secondary loss of expression from the intact allele may also occur owing to reduced *GATA2* occupancy at its own promoter.

† May result in undetectable transcription of one allele.

‡ May cause expression of protein with modified or dominant negative function.

Although more than half the variants described are single amino acid substitutions that may lead to the translation of mutated protein with altered function, there is reasonable expectation that the functional effects of heterozygous mutation are primarily due to haplo-insufficiency (Table I). The main argument is that gene deletions and frame-shift mutations that are null alleles, lead to virtually the same constellation of phenotypes as amino acid substitution variants. Many single amino acid substitutions are predicted to significantly impair DNA binding of the zinc fingers potentially making them functionally inactive (Dickinson *et al*, 2011). However, it is also possible that these mutants have residual function or can even act in a dominant negative fashion, as reported for T354M (Hahn *et al*, 2011). Gain of function is reported for the L359V variant found in blast transformation of chronic myeloid leukaemia (CML) (Zhang *et al*, 2008).

Of all the known regulatory regions of *GATA2*, only the intron 5 enhancer has been reported to contain germ-line mutations (Johnson *et al*, 2012; Hsu *et al*, 2013). This enhancer has been shown to be critical for *GATA2* expression in endothelium and HSC (Khandekar *et al*, 2007; Johnson *et al*, 2012; Lim *et al*, 2012; Gao *et al*, 2013). The existence of several patients with reduced GATA2 due to allelic expression imbalance suggests that other regulatory sites will be involved but sequencing the 5′ enhancers of *GATA2* has so far proven unfruitful (Hsu *et al*, 2013; unpublished observations). A recent report in sporadic AML invokes hypermethylation as a potential mechanism of loss of *GATA2* expression (Celton *et al*, 2014). Regulation of *GATA2* translation by MIR23A binding to the 3′-UTR has also recently been described as the mechanism by which SON protein enhances *GATA2* expression (Ahn *et al*, 2013).

## Clinical syndromes associated with *GATA2* mutation

The clinical syndromes of human GATA2 deficiency were uncovered by four independent groups, each working with a different focus. Monocytopenia with susceptibility to atypical mycobacterial infection, such as mycobacteriuma avium complex, was described as ‘monoMAC’ (Vinh *et al*, 2010) and *GATA2* mutation was revealed by a candidate sequencing approach (Hsu *et al*, 2011). Loss of dendritic cells (DCs), monocytes B and Natural Killer (NK) lymphoid cells (DCML deficiency) was described in four individuals (Bigley *et al*, 2011) all found to be harbouring *GATA2* mutation by exome sequencing (Dickinson *et al*, 2011). These studies were closely followed by two groups who had carefully curated cohorts of familial MDS/AML and hereditary lymphoedema with MDS (Emberger syndrome), ultimately localizing the genetic defect to *GATA2* (Scott *et al*, 2010; Hahn *et al*, 2011; Ostergaard *et al*, 2011). In all familial cases, the trait was inherited in an autosomal dominant fashion.

A succession of follow-up reports established new cases and recalled a fascinating series of historical precedents by retrospective diagnosis (Kaur *et al*, 1972; Robinson *et al*, 1983; Biron *et al*, 1989; Ballas *et al*, 1990; Couderc *et al*, 1992; Horwitz *et al*, 1996; Wendland *et al*, 2000; Khanjari *et al*, 2003; Witzke *et al*, 2004; Bodor *et al*, 2012; Holme *et al*, 2012; Ishida *et al*, 2012; Kazenwadel *et al*, 2012; Camargo *et al*, 2013; Mace *et al*, 2013; Mutsaers *et al*, 2013; Niimi *et al*, 2013; Pasquet *et al*, 2013; Chou *et al*, 2014; Gao *et al*, 2014; West *et al*, 2014). The first we are aware of was a report in this journal in 1972, describing an Icelandic family with MDS/AML in association with trisomy 8 and Pelger-Huet abnormality (Kaur *et al*, 1972), subsequently traced two generations later to a *GATA2* T354M mutation (Dickinson *et al*, 2014). Other familial cases of AML with immunodeficiency and pulmonary dysfunction have also since been tracked to *GATA2* mutation (Robinson *et al*, 1983; Horwitz *et al*, 1996). The original case of human NK deficiency is now known to be a GATA2 phenotype (Biron *et al*, 1989; Mace *et al*, 2013). *GATA2* mutation has also been identified in paediatric neutropenia and aplastic anaemia (Pasquet *et al*, 2013).

**Figure 3:**
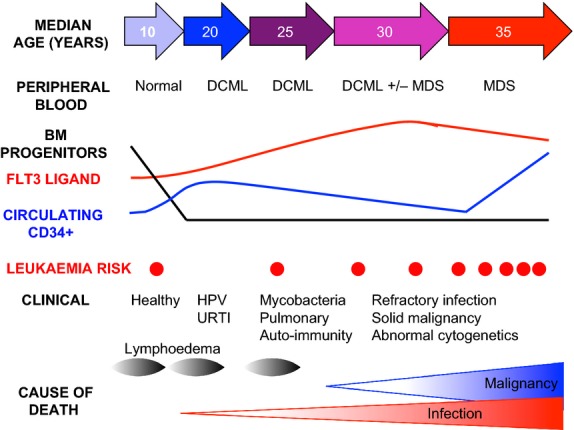
Schematic diagram summarizing the evolution of cellular deficiency in GATA2 mutation. Bone marrow multi-lymphoid progenitors are rapidly lost, even in healthy carriers. Peripheral blood CD34 counts are elevated in many patients and tend to decline with advancing disease. FLT3 ligand is progressively elevated but declines as patients develop MDS. A rapid rise in CD34^+^ cells and decline in FLT3 ligand may signify the onset of MDS or AML although AML may occur sporadically without prior cytopenia. HPV: human papilloma virus infections; URTI: recurrent bacterial upper respiratory tract infection; FLT3 ligand, fms-related tyrosine kinase 3 ligand; DCML, loss of dendritic cells, monocytes, B and Natural Killer lymphoid cells; MDS, myelodysplastic syndrome; AML, acute myeloid leukaemia.

The protean manifestations of *GATA2* mutation and clinical progression of patients have been documented more recently through two larger cohort studies drawn from North America and Europe, summarized in Table II and Fig3 (Dickinson *et al*, 2014; Spinner *et al*, 2014). Penetrance in these selected patients and their family members is more than 90% but with an extended age range of onset from 5 to 55 years and a median survival of 60 years. The most common features are warts from widespread human papilloma virus (HPV) infection, progression to MDS, pulmonary dysfunction including pulmonary alveolar proteinosis (PAP), infection with mycobacteria or fungi and lymphoedema. It is notable that many recognizable features of GATA2 deficiency were described in the early case reports.

**Table II:** Principal clinical features of *GATA2* mutation

Feature	Details	Approximate frequency
MDS/AML	Secondary mutations: See Table IV	30–50% at presentation 30-year median onset 90% lifetime risk
Warts, severe viral infection	HPV all serotypes Herpesviruses	60–70% at presentation 10–20% disseminated CMV, EBV or VZV
Pulmonary alveolar proteinosis or decreased lung function	PAP (GM-CSF antibody negative) Pulmonary artery hypertension Loss of volume or diffusion Pneumonia	18% proven PAP 10% PAH 50% abnormal PFT 14% pneumonia
Mycobacterial or fungal infection	NTM (MAC and others) Aspergillosis Histoplasmosis	20–50% NTM 16% apergillosis 9% histoplasmosis
Recurrent upper respiratory tract infection	Otitis Sinusitis	10–20%
Autoimmune manifestation	Panniculitis Arthritis Lupus-like Hypothyroidism Hepatitis/PBC	30% panniculitis up to 50% overall
Solid malignancy	HPV related Breast cancer Skin cancers EBV+ mesenchymal	20–35% intra-epithelial neoplasia 22% of women >35 years breast cancer other skin cancer 10%
Lymphedema	Childhood or adolescence	11–20%
Thrombosis	DVT PE Catheter-related	25% risk overall
Deafness	Congenital	20% abnormal audiograms
Preterm labour	Maternal trait	33%

MDS, myelodysplastic syndrome; AML, acute myeloid leukaemia; HPV, human papilloma virus; CMV, cytomegalovirus; EBV, Epstein–Barr virus; VZV, varicella zoster virus; PAP, pulmonary alveolar proteinosis; GM-CSF, granulocyte-macrophage colony-stimulating factor; PAH, pulmonary arterial hypertension; PFT, pulmonary function tests; NTM, nontuberculous mycobacteria; MAC, mycobacterium avium complex; PBC, primary biliary cirrhosis; DVT, deep vein thrombosis; PE, pulmonary embolism.

Several kindreds have been reported with unaffected individuals carrying *GATA2* mutation into their fifth and sixth decades but overall, the lifetime risk of MDS is approximately 90%. By the age of 60 years, the majority of patients will have had additional complications of defective cell-mediated immunity such as warts, herpes virus, mycobacterial or fungal infection. About 20% develop PAP and up to 50% have evidence of pulmonary dysfunction. Malignant disease is common, mostly due to HPV-driven intraepithelial neoplasia but an increase in breast cancer, squamous cell carcinoma and Epstein–Barr virus (EBV) positive neoplasms is reported. Initial reports linking frameshift or null mutations to a higher risk of lymphoedema are supported by larger studies (Hyde & Liu, 2011; Spinner *et al*, 2014). Severe viral infection is also significantly increased in these individuals. In contrast, MDS and AML appears to be an equal risk with all types of *GATA2* mutation (Dickinson *et al*, 2014; Spinner *et al*, 2014).

These cohort studies highlight first, that carriers are haematologically normal at birth; second, that the phenotype of mononuclear cytopenia, or DCML deficiency, evolves over time; and third, that loss of mononuclear cells is a common feature of all patients with symptoms (Dickinson *et al*, 2014; Spinner *et al*, 2014). In both studies, a simple clinical score of the 4–5 most frequent complications shows a strong association with progressive deterioration in cell counts. These findings explain the fact that most patients with *GATA2* mutation have unremarkable childhood vaccination and infection histories and normal levels of class-switched immunoglobulin and memory T cells, as young adults. From a haematological perspective, there are several precedents of hereditary bone marrow failure (BMF) syndromes presenting with an ‘accessory phenotype’, such as thrombocytopenia in *RUNX1* mutation and eosinophilia in *CEBPA* mutation (Owen *et al*, 2008; Carmichael *et al*, 2010). In *GATA2* mutation this accessory phenotype appears to be loss of mononuclear cells. It is conceivable that MDS or AML could develop without prior warning from bone marrow (BM) with constitutive *GATA2* mutation. However, most hereditary MDS/AML kindreds have younger generations with clear DCML deficiency and MDS and this may have been overlooked in their affected ancestors; parents were reported to have developed AML and deceased grandparents, simply ‘Acute Leukaemia’.

## GATA2 mutation and bone marrow failure

Previously reported associations between susceptibility to mycobacterial infection, PAP and MDS, foreshadow the discovery of *GATA2* mutation as a unifying cause. By the age of 60 years, 90% of patients will have developed refractory cytopenia and multilineage dysplasia that meets standard criteria and is often associated with the acquisition of additional genetic defects. Prior to this, the natural history of progressive BMF can be dissected in detail.

As patients with *GATA2* mutation progress, a number of features appear that make them readily distinguishable from sporadic MDS (Calvo *et al*, 2011; Dickinson *et al*, 2014) (Table III). Cardinal signs are family history of MDS or unusual haematological problem, young age of presentation (median age 21–33 years), atypical infection, severe monocytopenia and a high frequency of hypocellularity, megakaryocyte atypia and fibrosis in the BM. Compared with unselected MDS patients attending outpatients, patients with *GATA2* mutation have better preserved haemoglobin, neutrophil and platelet levels but much more severe deficits of mononuclear cells (Calvo *et al*, 2011; Dickinson *et al*, 2014; Spinner *et al*, 2014). Monocytopenia, if present, is highly discriminatory but it can be masked by progenitor or atypical lymphocyte expansion on routine blood counting. In this case, simple lymphocyte subset analysis will reveal striking B and NK deficiency. BM examination may be normal or reveal only slight hypocellarity and megakaryocyte atypia, even when there is gross mononuclear cell deficiency.

**Table III:** Differences between *GATA2* mutation and sporadic myelodysplasia

Variable	Normal	*GATA2* mutation	Sporadic MDS
Median age, years		21–33	66
Family history		1 in 3	Rare
Hb, g/l	115–145	115–123	96 (supported)
Neutrophils, ×10^9^/l	2–8	1·8–2·6	0·55
Platelets, ×10^9^/l	150–400	127–160	49
mDCs, ×10^9^/l	0·007–0·02	0	0·008
pDCs, ×10^9^/l	0·008–0·022	0	0·009
Monocytes, ×10^9^/l	0·31–0·56	0·01	0·1
B cells, ×10^9^/l	0·033–0·291	0·01	0·041
NK cells, ×10^9^/l	0·08–0·155	0·002	0·111
CD4:CD8 ratio	>1	<1	>1
BM cellularity		Hypocellular in 72%	Hypercellular in 95%
Fibrosis		73% of cases	10% of cases
Megakaryocytes		90% atypia	50–60% atypia
FLT3 ligand, pg/ml; median (range)	71 (56–141)	4752 (294–8750)	163 (57–613)
Infection		Warts, mycobacteria, herpesviruses	Neutropenic fever

Normal: values represent normal ranges. GATA2: range of median values from multiple publications or median values from Dickinson *et al* (2014). Sporadic MDS: median values from Dickinson *et al* (2014). mDCs, lineage negative HLA-DR^+^ CD1c^+^ population; pDCs, lineage negative HLA-DR^+^ CD123^+^ population; NK, natural killer; BM, bone marrow.

An interesting feature of *GATA2* mutation is the extremely high elevation of FLT3 ligand. This begins at an early stage and soon separates *GATA2* mutation from sporadic MDS. Progressive elevation of FLT3 ligand is associated with worsening cytopenias and clinical complications, suggesting that it may be useful in clinical monitoring (Bigley *et al*, 2011; Dickinson *et al*, 2014).

GATA2-deficient BM has been subjected to flow cytometry and clonality studies, revealing profound defects that are not apparent by routine morphological analysis, during early stages of the disease (Bigley *et al*, 2011; Calvo *et al*, 2011; Dickinson *et al*, 2014). In the CD34^+^ progenitor compartment there is complete absence of the primitive CD38− multi-lymphoid or lymphoid-primed multipotent progenitors [MLP/LMPP, as defined by various groups (Doulatov *et al*, 2010; Goardon *et al*, 2011)], and a severe depletion of CD38^+^ granulocytic monocytic progenitors, although sufficient remain to sustain neutrophils in many patients. Clonality, as tested by allele-specific polymerase chain reaction of X chromosome transcripts in females, appears as a relatively early sign, anticipating cellular deficiency. In the early stages of BMF, there is mobilization of CD34^+^ progenitors into the peripheral blood but these appear to fade with increasing hypocellularity and cytopenia before increasing again in the context of leukaemic transformation (Bigley *et al*, 2011; Dickinson *et al*, 2014). As patients progress, disordered myelopoiesis is evident from the lack of monocyte precursors, hypogranularity of myelocytes and lack of CD16 expression (Calvo *et al*, 2011).

The gradual and progressive decline in stem cell function in humans is reflected in the results of several model systems in which GATA2 is required to maintain stem cell self-renewal capacity (Tsai & Orkin, 1997; Nottingham *et al*, 2007; de Pater *et al*, 2013). The absence of MLP/LMPP, loss of lymphoid and monocyte/DC haematopoiesis, with relative sparing of erythropoiesis and granulopoiesis, resembles an accelerated ageing phenotype (Gekas & Graf, 2013). It will be of interest to determine whether objective signs of this can be detected prematurely in GATA2 patients (Bakker & Passegue, 2013).

The parallel failure of monocytopoiesis and lymphopoiesis highlights the inadequacy of conventional models of haematopoiesis in which the primary bifurcation in cell fate is a split between ‘myeloid’ and lymphoid’. Conventionally, monocytes and lymphocytes would arise from opposite lineages. It is now thought that monocyte potential and indeed granulocyte potential is retained by primitive multi-lymphoid lymphoid or lymphoid-primed progenitors (Goardon *et al*, 2011; Doulatov *et al*, 2012) and the first fate decision is more accurately defined as the separation of megakaryocyte and erythroid potential from ‘nucleated cell potential’ (Arinobu *et al*, 2007; Sanjuan-Pla *et al*, 2013). Megakaryocytes are closely related to HSCs and the platelet lineage, including platelets themselves, retains a high level of *GATA2* mRNA and GATA2 protein.

## *GATA2* mutation and immune dysfunction

The most common manifestations of immune dysfunction in *GATA2* mutation are generalized warts and mycobacterial infection. Recurrent respiratory tract infection and a miscellany of infections due to impaired cell-mediated immunity including EBV, herpes simplex virus, varicella zoster virus (VZV), invasive aspergillus, histoplasmosis and candida are also seen (Vinh *et al*, 2010; Camargo *et al*, 2013; Dickinson *et al*, 2014; Spinner *et al*, 2014). The combination of impaired viral clearance and defective immunosurveillance is presumably responsible for a high rate of malignant transformation of HPV-driven neoplasia and increased incidence of solid neoplasms. In addition to direct infectious complications, pulmonary alveolar proteinosis maybe exacerbated by recurrent respiratory and mycobacterial infections. It is difficult to tease out the precise mechanisms of immunodeficiency when so many components of innate and adaptive immune systems are compromised. Perhaps one of the most striking features of some patients with *GATA2* mutation is how little infection they experience despite very profound cellular deficiencies. This suggests that, while immunocompetence is critical in childhood, immunological memory is the main factor sustaining adult resistance to infection.

### Warts

It has been noted that few other conditions cause papillomatosis or generalized verrucosis to the same degree as GATA2 deficiency, namely Epidermodysplasia verruciformis (EV) warts, hypogammaglobulinemia, immunodeficiency, myelokathexis (WHIM) syndrome warts, immunodeficiency, lymphoedema and anogenital dysplasia (WILD) syndrome, DOCK8 deficiency syndrome and idiopathic CD4 lymphocytopenia (Vinh *et al*, 2010; West *et al*, 2014). These are easily distinguished on clinical grounds or routine investigation. The combination of warts with monocytopenia is highly suggestive of a diagnosis of *GATA2* mutation.

### Life without DCs

Lack of DCs was highlighted in the description of DCML deficiency (Bigley *et al*, 2011) but was also previously recorded in at least one case report (Witzke *et al*, 2004). The lack of DCs may impair recognition of viruses and intracellular pathogens contributing to disseminated herpes virus infection and mycobacterial susceptibility. In earlier case reports, defects in antigen-presenting cell-dependent mitogen responses (concanavalin A), responses to immunization, recall antigens and delayed type hypersensitivity are all documented (Kaur *et al*, 1972; Witzke *et al*, 2004). A systematic study of vaccine responses has not been performed. Interestingly, patients with undetectable blood (and probably also tissue DCs) still experience graft-*versus*-host disease when transplanted (Cuellar-Rodriguez *et al*, 2011). The persistence of tissue macrophages and epidermal Langerhans Cells may offer an alternative route of antigen presentation when DCs are profoundly depleted (Bigley *et al*, 2011).

### Monocytes and mycobacterial infection

Excluding generalized BMF, *GATA2* mutation and hairy cell leukaemia (HCL) are two conditions notable for monocytopenia. Monocytopenia in HCL is less severe than GATA2 deficiency but also known to confer a risk of mycobacterial infection of 4–9% (Thaker *et al*, 2001). Monocytopenia leads to a profound impairment of whole blood cytokine responses in tests used to screen for genetic susceptibility to mycobacterial infection. Both IL12 and γ-interferon (IFNγ) responses are blunted (Bigley *et al*, 2011). This might be a trivial result given the absence of monocytes, but can be argued that it is a useful reflection of the *in vivo* defect and how its magnitude compares with established molecular causes of mycobacterial susceptibility, such as IFNγ receptor mutations (Fischer, 2007). Resistance to mycobacterial infection is complex and the well-described IFNγ-IL12 axis is multiply compromised by the absence of NK cells and DCs, in addition to monocytes. Although tissue macrophages are present at sites of infection, organized granulomatous inflammation is deficient.

### B cells

CD38^+^ CD10^+^ B/NK precursors are not detectable in the BM (Bigley *et al*, 2011) and CD38^+^ CD27^−^ transitional B cells, the most recent BM emigrants, are absent or severely depleted in GATA2 deficiency (Chou *et al*, 2014; Dickinson *et al*, 2014). Naïve B cells are also lower while there is a relative enrichment of memory B cells and plasmablasts. These changes are clearly consistent with failing production and the accumulation of differentiated cells. The NIH group first noted the presence of plasma cells and relatively normal immunoglobulin (Ig) in most patients (Vinh *et al*, 2010), although IgA deficiency and hypogammaglobulinaemia presenting with recurrent sinusitis is reported (Chou *et al*, 2014). Plasma cells are found in the BM and in inflamed tissues, many with an abnormal CD56^+^CD19-phenotype (Calvo *et al*, 2011). *GATA2* mutation quite powerfully demonstrates the autonomy of plasma cells in maintaining anamnestic humoral immune responses.

### NK cells

The original report of human NK deficiency was a case of *GATA2* mutation and other primary NK deficiency in humans is actually very rare (Biron *et al*, 1989; Mace *et al*, 2013). NK-mediated restriction of virally infected or dysplastic targets is impaired, weakening immunosurveillance of papillomatosis and other malignant transformations. The most obvious and consistent feature is loss of the CD56^bright^ population of immature NK cells, analogous to transitional B cells (Mace *et al*, 2013; Dickinson *et al*, 2014). Remaining NK cells appear skewed toward a more mature phenotype with loss of NKG2A (KLRC1) and CD62L (SELL) and increased expression of killer cell immunoglobulin-like receptors (KIRs) (Dickinson *et al*, 2014). These features are quite variable between patients and may reflect viral infection history, although are not obviously connected to cytomegalovirus (CMV) serostatus. NK cells are known to contain some *GATA2* mRNA and surviving cells appear to have additional functional defects that are not restored by γ-interferon (IFNγ) therapy *in vivo* (Mace *et al*, 2013). A few patients with absent NK precursors appear able to maintain relatively good total NK counts. The mechanism of this is unknown but may indicate the development of ‘NK memory’ in response to chronic antigen exposure (Romee *et al*, 2012). Other innate lymphocytes, including CD161^+^ mucosal invariant T cells, are also depleted in *GATA2* mutation, but NKT cells are less affected (Dickinson *et al*, 2014).

### T cell abnormalities and premature immunosenescence

In contrast to the other lymphocyte subsets, T cells are relatively well-preserved in patients with *GATA2* mutation. Peripheral T cell homeostasis in adults is largely independent of BM-derived T cell precursors. Thus in patients with progressive BMF, T cells, like plasma cells and Ig, can be maintained for many years. Inversion of the CD4:CD8 ratio (to <1) is a crude sign that CD4 helper function is failing and chronic antigen stimulation is driving expansion of CD8 memory cells (Vinh *et al*, 2010; Ostergaard *et al*, 2011; West *et al*, 2014). This is supported by more detailed phenotyping of T cell subsets showing diminution of naïve cells and expansion of terminally differentiated CD8^+^ CD45RA^+^ effector cells (T_EMRA_ cells) with lower CD27, SELL (CD62L), CD38 and HLA-DR than healthy controls (Dickinson *et al*, 2014). Large granular lymphocytosis has been observed in GATA2 patients (Vinh *et al*, 2010; Spinner *et al*, 2014) and is the morphological correlate of TEMRA cell expansion (Clemenceau *et al*, 2008). Overall, the immunophenotype of GATA2 patients in all lymphoid compartments is strongly reminiscent of the pattern of terminal differentiation seen with advancing age and in chronic viral infections, such as CMV, hepatitis C and human immunodeficiency virus (Strindhall *et al*, 2013). Thus, a prematurely aged phenotype is seen both in peripheral immune cells and in the BM progenitor compartment.

### Macrophages, Langerhans cells and pulmonary alveolar proteinosis

The appearance of inflammatory macrophages in the absence of circulating monocytes was first noted by Vinh *et al* (2010). Macrophages were also reported in BM, lung and healthy skin of GATA2 patients (Bigley *et al*, 2011). In the epidermis, Langerhans cells survive, albeit in reduced numbers. Local proliferation of Langerhans cells is well-described, accounting for the independence of this population in mice (Merad *et al*, 2002). However, the presence of macrophages was more difficult to reconcile with conventional model of a mononuclear phagocyte system in which monocytes continuously give rise to tissue macrophages. Human HSC transplantation had already indicated that macrophages were considerably more stable than DCs (Haniffa *et al*, 2009) but full independence of macrophages from circulating monocytes was not anticipated. Models of tissue macrophage homeostasis have since been dramatically revised and their longevity and stability in the absence of monocytes is now well-established in murine experiments (Hashimoto *et al*, 2013).

Macrophage homeostasis is highly relevant to lung hygiene. Defects of alveolar macrophages lead to PAP through inadequate clearance of surfactant proteins (Carey & Trapnell, 2010). The primary cause of this rare condition is autoimmune antibodies to granulocyte-macrophage colony-stimulating factor (GM-CSF, CSF2), which impair the GM-CSF-mediated maturation of alveolar macrophages. Whole lung lavage and GM-CSF injection usually restores macrophage function in these cases. In contrast, patients with PAP due to *GATA2* mutation do not have anti-GM-CSF antibodies and respond poorly to lavage and GM-CSF therapy. Although they have an adequate number of alveolar macrophages, both in biopsies and lavage fluid, a cell-intrinsic effect of *GATA2* mutation appears to interfere with their functional maturation. GATA2 interacts with many signalling cascades through modulating the expression of key receptors or transduction proteins, such as the M-CSF (CSF1) receptor, phospholipase C and IL1 signalling pathway (Bigley *et al*, 2011; Ishijima *et al*, 2012; Wu *et al*, 2013). Protein-protein interactions are also reported with STAT proteins and SMAD (Ezoe *et al*, 2005; Dong *et al*, 2014) as well as direct effects on phagocytosis (Lasbury *et al*, 2003). The potential functions of GATA2 outside the nucleus are only recently recognized and further studies are required to elucidate the mechanism of PAP in GATA2 deficiency.

## Thrombosis

GATA2 is highly expressed in megakaryocytes, platelets and vascular endothelium (Johnson *et al*, 2012; Lim *et al*, 2012) and thrombosis is reported in up to 25% of patients (Spinner *et al*, 2014). Lupus anticoagulant, infection and malignancy add further to intrinsic risks and patients may develop refractory problems with thrombosis, cellulitis and soft tissue infections.

## Autoimmune manifestations

Patients with *GATA2* mutation experience a spectrum of autoimmune disease. Panniculitis, either as isolated inflammatory nodules or more classical erythema nodosum are possibly the most common but arthritis, lupus-like syndromes autoimmune hepatitis and primary bilary cirrhosis have also been described (Dickinson *et al*, 2014; Spinner *et al*, 2014). A severe deficit of T_regs_, was reported in DCML deficiency (Bigley *et al*, 2011). Although it could be argued that this was secondary to DC deficiency, a phenomenon reported in mice (Darrasse-Jeze *et al*, 2009), it is likely that widespread failure of mononuclear cell production is at least partly to blame in *GATA2* mutation. In the B cell compartment, the CD38^−^CD21^−^ population, previously reported to be associated with autoimmunity, is increased in some GATA2 patients (Dickinson *et al*, 2014).

## Solid malignancy

Premature death has occurred in a number of patients due to solid malignancy. In keeping with the prevalence of HPV infection, anogenital intraepithelial dysplasia is a serious concern and squamous cell carcinoma occurs with higher frequency than expected (Dickinson *et al*, 2014; Spinner *et al*, 2014). EBV-related mesenchymal tumours, adenocarcinoma, desmoid tumour of the chest wall and schwannoma have also been reported (Dickinson *et al*, 2014; Spinner *et al*, 2014).

## Lymphedema, deafness, congenital anomalies and preterm labour

Lymphoedema and myelodysplasia are the primary features of Emberger syndrome (Ostergaard *et al*, 2011). Deafness is also seen especially in Emberger syndrome and occurs in association with whole gene deletions of *GATA2* (Ostergaard *et al*, 2011; Kazenwadel *et al*, 2012) possibly due to failure of generation of the perilymphatic space surrounding the semi-circular canals (Haugas *et al*, 2010). The evolution of lymphoedema is puzzling. Unlike most congenital forms of lymphoedema, which occur bilaterally as a child begins to walk, patients with *GATA2* mutation often describe a precipitating event occurring later and leading to unilateral limb swelling. It has been elegantly demonstrated that *GATA2* is expressed in endothelial cells and lymphatic valves (Kazenwadel *et al*, 2012; Lim *et al*, 2012) but it is also conceivable that the BM-dependent development or maintenance of lymphoid tissue is additionally culpable. Suggestions that N-terminal frameshift mutations or larger deletions of *GATA2* are more likely to cause lymphoedema and non-haematopoietic defects are supported by the larger cohort studies (Spinner *et al*, 2014).

As initially noted by Vinh *et al* (2010), premature labour is often experienced by females with *GATA2* mutation (Dickinson *et al*, 2014; Spinner *et al*, 2014). The problem appears to be maternal rather than fetal as it affects wild-type fetuses of mothers with mutations but not fetuses with *de novo* mutations. There are many potential explanations for premature labour including roles for GATA2 in the uterus and placenta (Ma *et al*, 1997; Rubel *et al*, 2012). In a single case, successful BM transplantation allowed the patient to carry a second pregnancy to term (unpublished observations).

## Acquired genetic abnormalities and evolution to leukaemia

Hereditary MDS/AML, rather than immune dysfunction, is the principle clinical feature of several kindreds with *GATA2* mutation (Hahn *et al*, 2011; Bodor *et al*, 2012; Holme *et al*, 2012; Ishida *et al*, 2012; Fujiwara *et al*, 2014). MDS/AML is the presenting feature of 30–50% of cases and the actuarial risk of developing MDS/AML by 60 years of age is close to 90% (Dickinson *et al*, 2014; Micol & Abdel-Wahab, 2014; Spinner *et al*, 2014). Initially, it was thought that point mutation of the second zinc finger, such as T354M, might confer an increased risk of leukaemic transformation over frameshift mutations or null alleles but this is not borne out by larger cohort studies (Dickinson *et al*, 2014; Spinner *et al*, 2014). Constitutive genetic background may have an influence on the risk of leukaemic transformation and susceptibility to infection although it is notable that a range of clinical phenotypes can be seen in different individuals within one pedigree (Holme *et al*, 2012; Mutsaers *et al*, 2013; Spinner *et al*, 2014).

The acquisition of additional genetic abnormalities in the transformation of *GATA2* mutation to multilineage dysplasia is clearly presaged by the high incidence of monosomy 7 and trisomy 8 in familial cases of MDS/AML (Hahn *et al*, 2011; Ostergaard *et al*, 2011; Bodor *et al*, 2012; West *et al*, 2013; Dickinson *et al*, 2014; Micol & Abdel-Wahab, 2014; Spinner *et al*, 2014). Recently, acquired mutation of ASXL1 (chr 20q11) has been demonstrated in approximately 30% of individuals with *GATA2* mutation evolving to MDS. Acquired *ASXL1* mutation is strongly associated with the presence of monosomy 7, BM hypercellularity and chronic monomyelocytic leukaemia (Bodor *et al*, 2012; West *et al*, 2013; Micol & Abdel-Wahab, 2014). Whole genome sequencing in one patient has also identified mutations in *EZH2*, *HECW2* and *GATA1* (Fujiwara *et al*, 2014); the spectrum of somatic mutations that are known to occur with germline *GATA2* mutation is summarized in Tables IV and SI. The presence of monosomy 7, *ASXL1* mutation and trilineage dysplasia are all high risk features in the biogenesis of AML (West *et al*, 2013). A number of patients with *GATA2* mutation have received successful haematopoietic stem cell transplantation, precisely because a high risk AML was detected, according to standard criteria. The detection of a *GATA2* germ-line mutation does not appear to mitigate the risk of AML that follows, whatever the subsequent genetic events (Cuellar-Rodriguez *et al*, 2011; Dickinson *et al*, 2014; Spinner *et al*, 2014).

**Table IV:** Genetic abnormalities associated with *GATA2* mutation and nature of haematological transformation

*GATA2* configuration	Associated with	Outcome
Germline heterozygous mutation	***ASXL1*** ***monosomy 7*** ***trisomy 8*** *trisomy 21* *der(1;7), +1q −7q* *EZH2* *HECW2* *GATA1*	High risk MDS/AML
Somatic heterozygous mutation (often ZF1; also ZF2)	**bi-*CEBPA*** m-*CEBPA* *t(15;17)* *NPM1*	Favourable risk AML
	*RUNX1, IKZF1, NRAS, KRAS, FLT3-*ITD*, KIT, WT1, FBXO3, MLF1IP, STT3B, IDH1, DNMT3A*	Intermediate-high risk AML
Somatic heterozygous mutation (often ZF2)	**t(9;11) Philadelphia** *ANO5, MAX, ENO1, COL3A1, AFP, SERPINA1, MGAT5B, ZNF208*	Blast crisis CML (High risk AML)
Transposition of *GATA2* distal haematopoietic enhancer (G2DHE; −110 kb)	**inv(3)/t(3;3)** Activation of EVI-1 by G2DHE monosomy 7	High risk MDS/AML

ZF, zinc finger; G2DHE, *GATA2* distal haematopoietic enhancer; bi, bi-allelic; m, mono-allellic; AML, acute myeloid leukaemia; CML, chronic myeloid leukaemia; MDS, myelodysplastic syndrome.

Bold in column 2 represents the mutations most commonly associated with the corresponding GATA2 mutation in column 1.

The knowledge that *GATA2* mutation is a constitutive risk factor for MDS/AML begs an important question of whether acquired *GATA2* mutation is among the key leukaemia-initiating events in sporadic MDS/AML (Table IV). The incidence in unselected cases of MDS or AML is actually quite low, at <5%, and may include cases of *GATA2* germ-line mutation that were assumed to be somatic, in the absence of a germ-line DNA control (Yan *et al*, 2011; Luesink *et al*, 2012; Papaemmanuil *et al*, 2013; Shiba *et al*, 2014). Gain of function mutation L359V has been documented in blast transformation of CML, associated with typically poor outlook (Zhang *et al*, 2008, 2009). In contrast, a high level of mutation (approximately 40%) is observed with bi-allelic mutation of *CEBPA*, conferring a better prognosis than *CEBPA* mutation with wild-type *GATA2* (Greif *et al*, 2012; Fasan *et al*, 2013; Green *et al*, 2013; Grossmann *et al*, 2013; Shiba *et al*, 2014). Excluding those cases with *FLT3*-internal tandem duplication (ITD) (which associates with *GATA2* wild-type) may lessen the observed beneficial effect of a *GATA2* mutation (Green *et al*, 2013).

Although convincing evidence of recurrent mutation of *GATA2* was not originally found in sporadic AML, it was first noted more than 10 years ago, that the distal 5′ regulatory elements of *GATA2*, were involved in chromosome 3q21 rearrangements and that dysregulation of *GATA2* expression might contribute to leukaemogenesis (Zhou *et al*, 1998; Wieser *et al*, 2000). This hypothesis has recently been elegantly confirmed, with the demonstration that 3q rearrangement is a double hit disease involving simultaneous removal of the *GATA2* distal haematopoietic enhancer (G2DHE) and *cis*-activation of the neighbouring locus EVI1 (Groschel *et al*, 2014; Yamazaki *et al*, 2014). Thus loss of *GATA2* expression is closely involved in leukaemogenesis involving 3q rearrangements. The risk profile of MDS/AML associated with *GATA2* mutation thus depends upon the germline configuration of *GATA2* and the sequence and location of associated genetic events (Table IV). Somatic monosomy 7 confers a high risk phenotype upon MDS/AML arising with germline *GATA2* mutation, while somatic mutation of *GATA2* with pre-existing bi-allelic *CEBPA* mutation is favourable risk. Other pre-existing mutations are intermediate or high risk (e.g. *FLT3*-ITD, *RAS* mutations and *WT1* mutation). The double jeopardy of somatic inv3 or t(3;3) in reducing GATA2 but co-activating EVI1 is undoubtedly high risk.

## Clinical management of individuals with *GATA2* mutation

Recognition of the clinical syndromes of GATA2 deficiency and genetic diagnosis have facilitated the rapid identification of more than 200 individuals with *GATA2* mutation (Dickinson *et al*, 2014; Spinner *et al*, 2014). Cross-sectional analysis of these is beginning to inform clinical management. A continuing challenge is the recognition of new cases that may present with a spectrum of manifestations to haematologists, infectious disease physicians, dermatologists, chest physicians, rheumatologists and many other specialties. Monocytopenia remains the most vital clue to the haematologist making a remote diagnosis. Suggested follow up investigations are listed in Table V.

**Table V:** Investigation of GATA2 deficiency

History and examination
Personal history, sometimes with family history of warts, mycobacterial infection, autoimmunity, chest disease, cytopenias, acute myeloid leukaemia
Routine tests
*Blood count*: Monocytopenia (may be obscured by progenitor mobilization or atypical lymphocytosis during infection)
*Lymphocyte subsets*: B cell and NK cell deficiency; CD4:CD8 inversion (<1·0); absence of CD1c^+^, CD141^+^ and plasmacytoid blood DCs
*Immunoglobulins* normal levels, occasional IgA or IgG deficiency
*Bone marrow*: megakaryocyte dysplasia, hypocellularity, fibrosis; cytogenetics: monosomy 7; trisomy 8; *ASXL1* mutation
Further investigations
*Blood*: Lupus anti-coagulant; elevated FLT3 ligand (10- to 100-fold); absent transitional B cells and CD56^bright^ NK cells
*Bone marrow Flow cytometry*: loss of primitive MLP, LMPP population and reduction of GMP; CD56^+^ plasma cells present (all morphological tests may be normal)
Lungs: diminished lung volumes and transfer factor; pulmonary infilatrates on CT; pulmonary alveolar proteinosis on biopsy without GM-CSF antibodies
Tissues biopsy: special stains for mycobacteria and fungi; neoplastic lesions investigated for HPV and herpes virus nucleic acid or antigens
Confirmatory test
*GATA-2* gene deletion, mutation in codons 1–398 or intron 5 enhancer

MLP, multi-lymphoid progenitors; LMPP, lymphoid-primed multipotent progenitors; GMP, granulocytic monocytic progenitors; CT computerized tomography.

With family members being diagnosed through genetic testing, it is important that this is performed with appropriate counselling. GATA2 deficiency and its definitive treatment are significant health issues. An additional complication is the pressure upon siblings to be screened as potential HSC donors. Their right to refuse genetic screening needs to be considered prior to tissue typing to prevent undue coercion.

With a median survival of 60 years (Spinner *et al*, 2014), a watch and wait policy is acceptable for many patients. Warts vary in severity but can be difficult to treat. It is prudent to treat respiratory infection promptly and to maintain vigilance for mycobacterial infection. Patients will benefit from serial pulmonary function testing, computerized tomography scanning and, possibly, prophylactic azithromycin. Panniculitis can be painful and responds to steroids, although steroid-sparing agents, such as dapsone, are preferable. The use of corticosteroids is difficult as blood counts, chest symptoms and inflammatory problems may respond promptly but caution is advisable in view of the level of subclinical immunoparesis. Serum electrophoresis should be performed and immunoglobulin replaced if necessary (Chou *et al*, 2014). Patients should have access to urgent medical care if necessary and should be under review by a haematologist.

Serial monitoring of peripheral blood leucocytes is useful and annual BM examination for morphology, flow cytometry and cytogenetics is advisable. FLT3 ligand, although not a routine test, shows a good correlation with clinical and cellular progression. *ASXL1* mutation screening of blood or BM samples is likely to be highly informative for the risk of leukaemic transformation.

Haematopoietic stem cell transplantation has been performed on at least 30 patients worldwide and has a good outcome (Cuellar-Rodriguez *et al*, 2011; Spinner *et al*, 2014). The development of MDS or AML, often with high risk cytogenetic features, has been an automatic trigger for many patients (most of whom were diagnosed with *GATA2* mutation retrospectively). The decision to move to transplant should not be delayed if cytogenetic abnormalities arise or there is a life-threatening complication at any stage. Although quite advanced PAP responds impressively (Cuellar-Rodriguez *et al*, 2011), salvaging patients in this situation is not an ideal strategy that should be obviated with genetic diagnosis and prospective monitoring. HPV infection responds well to transplantation and reversal of stage III intra-epithelial neoplasia, obviating potentially disfiguring surgery, has occurred over 12–18 months, possibly aided by HPV vaccination (unpublished observations). Close liaison with other specialist services and vigilance for monocytopenia will always be required to avoid the inadvertent progression of patients with unusual presentations, beyond the hope of cure. In the future, familial *GATA2* mutation may be an ideal opportunity to test gene therapy strategies as it is predicted that correction of haplo-insufficiency would lead to a competitive survival advantage for HSC at the BM niche.

## Supporting Information

**Table SI.** Reported mutations of GATA2 in humans.
