# Supplementary material for: Haematopoietic and immune defects associated with GATA2 mutation
Source: Br J Haematol. 2015 Feb 23;169(2):173–87. doi: 10.1111/bjh.13317 (PMC4409096; doi:10.1111/bjh.13317)
Supplement: Supplementary file 1 — Table SI. Reported mutations of GATA2 in humans. [file bjh0169-0173-sd1.docx]

### Supplementary Table 1. Reported mutations of GATA2 in humans

Abbreviations:

biGENE: bi-allelic mutation of GENE

mGENE: mono-allelic mutation of GENE

Regions of the GATA2 gene are colour-coded as follows:

Transactivation domain: purple

Negative regulatory domain: orange

Zinc finger 1: blue

Zinc finger 2: green

* Presumed somatic mutation because patient reported with sporadic leukaemia with no accessory haematological or immunodeficiency phenotype but remission blood or other source of germline DNA not sequenced for confirmation

+ Presumed germline mutation due to early onset or family history of accessory haematological or immunodeficiency phenotype but family member, remission blood or other source of germline DNA not sequenced for confirmation

^1^MutationTaster software predicts that the mutation will result in a null allele due to nonsense-mediated mRNA decay

^2^Functional assays and/or expression analysis suggest that the mutation is likely to result in a partial loss of function

^3^Functional assays and/or expression analysis suggest that the mutation is likely to result in complete loss of function.

^4^Mutation affects regulation of GATA2 expression and may result in no expression of one allele.

^5^Functional assays and/or expression analysis suggest that the mutation may cause dominant-negative function.

^6^Functional assays and/or expression analysis suggest that the mutation is likely to result in gain of function.

^7^Polyphen2 software prediction

| **Position**  **(amino acid)** | **Germline**  **or somatic** | **Consequence of mutation** | **Clinical Features**  **(associated mutations)** | **No. of cases** | **References** |
| --- | --- | --- | --- | --- | --- |
| Inv(3)/t((3;3) | Somatic^*^ | Null allele^4^  Activation of EVI1 | High risk AML (-7) | 36 (36 pedigrees) | Groschel et al., 2014  Yamazaki et al., 2014 |
| Whole gene deletion | Germline^+^  Germline^+^ | Null allele^1^ | Emberger’s; immunodeficiency; developmental delay; neutropenia;  MDS (-7); MDS (+21)  MDS (-7) | 3 (3 pedigrees)  1 | Kazenwadel et al., 2011  Pasquet et al., 2013 |
| M1del290 | Germline | Null allele^1^  Reduced IKBKG, CXCL12, SRC, NOTCH1 & FERMT3 expression  Increased RUNX1, FYN & ETS1 expression | MonoMAC, Emberger,  MDS/AML (-7, +8) | 2 (1 pedigree) | Hsu et al., 2011 |
| H26P | Germline | Probably damaging^7^  (potential ubiquitination site) | DCML/MonoMAC, MDS evolving to T cell NHL  (-7; biGATA2 with G28fs mutation) | 2 (1 pedigree) | Mutsaers et al., 2013 |
| G28fs | Germline | Null allele^1^ | DCML/MonoMAC, MDS evolving to T cell NHL  (-7; biGATA2 with H26P mutation) | 2 (1 pedigree) | Mutsaers et al., 2013 |
| P41A | Germline  SNP: rs143590990 | Probably damaging^7^  (potential ubiquitination site) | MDS | 4 (1 pedigree) | Holme et al., 2012 |
| R78fs | Germline | Null allele^1^ | Emberger, Emberger with MDS/AML  (-7) | 3 ( 1 pedigree) | Ostergaard et al., 2011 |
| G81fs | Germline^+^ | Null allele^1^ | MonoMAC, MDS | 1 | Hsu et al., 2011 |
| C85fs | Germline^+^ | Null allele^1^ | DCML, MDS  (-7) | 1 | Dickinson et al., 2013 |
| G101fs | Germline^+^ | Null allele^1^ | MonoMAC, MDS/AML to CMML  (-7, +8, ASXL1 ) | 1 | Hsu et al., 2013  West et al., 2014 |
| L105fs | Germline | Null allele^1^ | Emberger with MDS/AML (-7), Emberger, Emberger with MDS/AML | 2 (1 pedigree)  5 (1 pedigree; same as Holme et al., 2012) | Holme et al., 2012  Ostergaard et al., 2011 |
| S106fs | Germline | Null allele^1^ | DCML, lymphoedema | 2 (1 pedigree) | Dickinson et al., 2013 |
| S129X | Somatic^*^ | Null allele^1^ | AML [inv(9); t(15;17)] | 1 | Shiba et al., 2013 |
| V140fs | Germline^+^ | Null allele^1^ | Severe Infections, MDS  (ASXL1) | 1 | Spinner et al., 2013  West et al., 2014 |
| A194fs | Germline^+^ | Null allele^1^ | Emberger, AML  (-7) | 1 | Ostergaard et al., 2011 |
| A198fs | Germline^+^ | Null allele^1^ | DCML | 1 | Dickinson et al. unpublished |
| G199fs | Germline^+^ | Null allele^1^ | MonoMAC, MDS  (ASXL1) | 1 | Hsu et al., 2013  West et al., 2014 |
| G199fs | Germline | Null allele^1^ | DCML, MDS, lymphedema | 3 (1 pedigree) | Dickinson et al., 2013 |
| G200fs | Germline | Null allele^1^  Reduced GATA2 expression | DCML, MDS  (+8) | 3 (2 pedigree) | Dickinson et al., 2013 |
| A203P | Somatic^*^ | Possibly damaging^7^  (potential ubiquitination site) | AML (mCEBPA) | 1 | Green et al., 2013 |
| R204X | Germline^+^ | Null allele^1^ | Neutropenia, MonoMAC, AML [der(9)t(1;9)] | 1 | Pasquet et al., 2013 |
| E224X | Germline^+^ | Null allele^1^ | Neutropenia, warts | 1 | Pasquet et al., 2013 |
| P245fs | Germline^+^ | Null allele^1^ | DCML | 1 | Dickinson et al., 2013 |
| P254L | Germline^+^  ClinVar: rs387906630 | Probably damaging^7^  (potential ubiquitination site) | MonoMAC (potential biGATA2 with unknown mutation leading to no expression on other allele) | 1 | Hsu et al., 2011 |
| H258fs | Somatic^*^ | Null allele^1^ | AML (mCEBPA) | 1 | Green et al., 2013 |
| D259fs | Germline^+^ | Null allele^1^ | MonoMAC, MDS (-7) | 1 | Hsu et al., 2011 |
| G273X | Germline^+^ | Null allele^1^ | DCML, MDS | 1 | Dickinson et al. unpublished |
| R293Q | Somatic^*^ | Normal GATA2 expression | AML (biCEBPA; biGATA2 with A318T) | 1 | Greif et al., 2012 |
| P304H | Somatic^*^ | Altered ZF1 domain | AML | 1 | Yan et al., 2011 |
| R307L | Somatic^*^ | Altered ZF1 domain | AML (biCEBPA) | 1 | Fasan et al., 2013 |
| R307W | Somatic^*^ | Altered ZF1 domain | AML (biCEBPA) | 1 | Green et al., 2013 |
| R308P | Somatic^*^  Somatic^*^  Somatic^*^ | Delayed granulocytic differentiation  No effect on DNA binding and transcriptional activities^2^ | AML (biCEBPA)  AML (biCEBPA)  AML | 1  2 (2 pedigrees)  1 | Fasan et al., 2013  Green et al., 2013  Niimi et al., 2013 |
| N317fs | Germline^+^ | Null allele^1^ | MonoMAC | 1 | Hsu et al., 2011 |
| N317S | Somatic | Likely to alter DNA binding | AML (biCEBPA) | 1 | Fasan et al., 2013 |
| N317l | Somatic^*^  Somatic^*^ | Likely to alter DNA binding | AML (biCEBPA)  AML (biCEBPA) | 1  1 | Fasan et al., 2013  Green et al., 2013 |
| N317H | Somatic^*^ | Likely to alter DNA binding | AML (biCEBPA; biGATA2 with A318T) | 1 | Greif et al., 2012 |
| A318V | Somatic  Somatic^*^  Somatic^*^  Somatic^*^ | Likely to alter DNA binding | AML (biCEBPA)  AML (biCEBPA)  AML (biCEBPA)  AML (biCEBPA) | 3 (3 pedigrees)  3 (3 pedigrees)  1  1 | Fasan et al., 2013  Green et al., 2013  Greif et al., 2012  Luesink et al., 2012 |
| A318T | Somatic^*^  Somatic^*^  Somatic^*^  Somatic^*^ | Normal GATA2 expression  Likely to alter DNA binding  Reduced transcriptional activation of TCRδ  Reduced capacity to enhance CEBPA dependent activation of transcription^3^ | AML (mCEBPA)  AML (biCEBPA)  AML (biCEBPA)  AML (biCEBPA, DNMT3A) | 1  2 (2 pedigrees)  2 (2 pedigrees)  1 (1 pedigree) | Green et al., 2013  Greif et al., 2012 |
| A318G | Somatic^*^ | Likely to alter DNA binding | AML (biCEBPA) | 1 | Greif et al., 2012 |
| A318fs | Germline^+^ | Null allele^1^ | MonoMAC, MDS (-6, ASXL1) | 1 | West et al., 2014 |
| C319insR | Somatic | In-frame insertion  Altered ZF1 domain | AML (mCEBPA, FBXO3, MLF1IP, STT3B) | 1 | Yan et al., 2011 |
| G320D | Somatic^*^  Somatic^*^ | Normal GATA2 expression  May alter attachment of an adjacent β-hairpin loop that provides additional DNA binding contacts  Enhanced transcriptional activation of TCRδ  Reduced capacity to enhance CEBPA dependent activation of transcription^2^ | AML (biCEBPA)  AML (biCEBPA) | 1  3 (3 pedigrees) | Fasan et al., 2013  Greif et al., 2012 |
| G320V | Somatic^*^ | May alter attachment of an adjacent β-hairpin loop that provides additional DNA binding contacts | AML (biCEBPA) | 1 | Green et al., 2013 |
| L321R | Somatic^*^  Somatic^*^ | Likely to alter DNA binding | AML (biCEBPA)  JMML (-7, KRAS) | 1  1 | Fasan et al., 2013  Stieglitz et al., 2014 |
| L321F | Somatic  Somatic  Somatic^*^  Somatic^*^ | Normal GATA2 expression  Likely to alter DNA binding  Reduced transcriptional activation of TCRδ  Reduced capacity to enhance CEBPA dependent activation of transcription^3^ | AML (biCEBPA)  AML (RUNX1, IDH1G105)  AML (biCEBPA)  AML (biCEBPA) | 4 (4 pedigrees)  1 (1 pedigree)  1  5 (4 pedigrees) | Fasan et al., 2013  Fasan et al., 2013  Greif et al., 2012  Green et al., 2013 |
| L321H | Somatic | Likely to alter DNA binding | AML (biCEBPA; biGATA2 with L379Q) | 1 | Fasan et al., 2013 |
| L321P | Somatic^*^  Somatic^*^ | Likely to alter DNA binding | AML (mCEBPA)  AML (biCEBPA) | 1  1 | Green et al., 2013  Greif et al., 2012 |
| L321V | Somatic^*^  Somatic  Somatic | Likely to alter DNA binding | AML (biCEBPA)  AML (biCEBPA, IKZF1, KRAS)  AML (biCEBPA) | 1 (1 pedigree)  1 (1 pedigree)  1 | Greif et al., 2012  Fasan et al., 2013 |
| Q328P | Somatic^*^ | May affect the backbone fold of ZF1 and indirectly  alter DNA binding by perturbing the adjacent R330 or alternatively is involved in interaction with other domains  such as ZF2 | AML (biCEBPA) | 1 | Greif et al., 2012 |
| R329Q | Somatic^*^ | Altered ZF1 domain | AML (biCEBPA) | 1 | Fasan et al., 2013 |
| R330P | Somatic^*^ | Likely to alter DNA binding | AML (biCEBPA) | 1 | Green et al., 2013 |
| R330Q | Somatic^*^  Somatic^*^ | Likely to alter DNA binding | AML (biCEBPA)  AML (biCEBPA) | 2 (2 pedigrees)  2 (2 pedigrees) | Green et al., 2013  Greif et al., 2012 |
| R330L | Somatic^*^ | Likely to alter DNA binding | AML (biCEBPA)  AML (mCEBPA) | 1  1 | Green et al., 2013 |
| R330X | Germline  Germline^+^  Germline  Germline | Null allele^1^  Altered DNA binding  Reduced GATA-2 chromatin occupancy | Neutropenia, AML[der(1;7), +1q, -7q]  JMML (-7, KRAS)  MonoMAC, MDS (+8), lymphedema  Immunodeficiency and evolving MDS/AML (+8,  EZH2, HECW2, GATA1) | 5 (1 pedigree)  1  2 (1 pedigree)  1 | Pasquet et al., 2013  Stieglitz et al., 2014  Spinner et al., 2013  Fujiwara et al., 2014 |
| L332fs | Germline^+^ | Null allele^1^ | Emberger, MDS/AML  (-7, +8) | 1 | Kazenwadel et al., 2011 |
| R337X | Germline^+^  Germline^+^ | Null allele^1^  Reduced IKBKG and FERMT3 expression  Increased RUNX1 expression | Emberger with MDS/AML (-7)  Viral infections, lymphedema, MDS (-7, ASXL1) | 1  1 | Ostergaard et al., 2011  West et al., 2014 |
| L339fs | Germline^+^ | Null allele^1^ | DCML | 1 | Dickinson et al., unpublished |
| Intron variant:  1017+512del28 | Germline | Reduced GATA2 expression  Deletion of the E-box and disruption of the E-box/GATA composite element  Reduced IKBKG and FERMT3 expression  Increased RUNX1 expression^4^ | MDS, MonoMAC | 3 (1 pedigree) | Hsu et al., 2013 |
| Intron variant:  1017+572C>T | Germline | Reduced GATA2 expression  Disruption of ETS motif Reduced IKBKG and FERMT3 expression  Increased RUNX1 expression^4^ | MDS (-7), MonoMAC, MDS (ASXL1), LGL, CMML | 8 (4 pedigrees) | West et al., 2014 |
| c.1017+2T>G | Germline^+^ | Splice site changes  Potentially altered ZF1 & ZF2 domain | MonoMAC, MDS, AML | 1 | Kazenwadel et al., 2011 |
| Δ340-381 | Germline | In-frame deletion  Splice site changes  Deleted ZF2 domain  Reduced GATA2 expression^3^ | DCML | 1 | Dickinson et al., 2011 |
| Δ340-381 | Germline^+^  Germline^+^ | In-frame deletion  Splice site changes  Deleted ZF2 domain | DCML, MDS  MonoMAC, MDS [t(1;22), ASXL1], LGL | 1  1 | Dickinson et al., 2013  West et al., 2014 |
| A341fs | Germline^+^  Germline^+^ | Null allele^1^  Reduced GATA2 expression | DCML, lymphedema, developmental delay  Emberger, developmental delay | 1  1 | Dickinson et al., 2013  Ostergaard et al., 2011 |
| A341fs | Germline^+^ | Null allele^1^ | Emberger, developmental delay, MDS/AML | 1 | Ostergaard et al., 2011 |
| Δ341-346 | Somatic | In-frame deletion  Reduced transcriptional activation of CD34  Inhibited the ability of PU.1 to activate CSF1R.  Mild inhibited myelomonocytic differentiation and proliferation^2^ | CML with blast crisis (-19) | 1 | Zhang et al., 2008 |
| A342T | Germline | Altered ZF1 domain | AML (NRAS) | 3 | Shiba et al., 2013 |
| A342fs | Germline^+^ | Null allele^1^ | NK cell deficiency, aplastic anaemia | 1 | Mace et al., 2013 |
| C349G | Germline^+^ | Altered ZF2 domain | DCML | 1 | Dickinson et al., unpublished |
| A350_N351ins8 | Somatic^*^ | Reduced GATA2 expression  Reduction in DNA binding and activation of CD34 transcription  Inhibited granulocytic differentiation^3^ | AML | 1 | Niimi et al., 2013 |
| T354K | Somatic | May alter the ZF2 structure by affecting zinc contacts | AML (NPM1, NRAS, WT1) | 1 | Fasan et al., 2013 |
| T354M | Germline  Germline  Germline  Germline  Germline | Reduced GATA2 expression  Alters the ZF2 structure by affecting zinc contacts  Reduced IKBKG, CXCL12, SRC, NOTCH1 & FERMT3 expression  Increased RUNX1, FYN & ETS1 expression  Reduced DNA binding  Reduced transcriptional activation of RUNX1 and CD34  Inhibited the ability of PU.1 to activate CSF1R transcription  Inhibited granulocytic differentiation^5^ | DCML, MDS (+8), MDS  MDS (-7, ASXL1)  MDS (-7, const. 9p21-  22 variation), AML (const. 9p21-  22 variation), MDS (+8), AML [-7, +8, t(1q:7p)], MDS (-7), MDS, AML  MDS, AML  MonoMAC, MDS (+8), MDS (-7), MDS/AML, immunodeficiency, LGL | 6 (2 pedigrees)  9 (1 pedigree)  34 (3 pedigrees)  4 (1 pedigree)  5 (4 pedigrees) | Dickinson et al., 2013  Bodor et al., 2012  Hahn et al., 2011  Holme et al., 2012  Spinner et al., 2013 |
| 355delT | Germline | Shortens the conserved threonine string and may affect L359 which directly contacts the DNA  Reduced DNA binding  Reduced transcriptional activation of RUNX1 and CD34  Inhibited the ability of PU.1 to activate CSF1R transcription^5^ | Familial early onset MDS and/or AML (+8, -7) | 2 (1 pedigree) | Hahn et al., 2011 |
| T357S | Somatic | Altered ZF2 domain | AML [+6, +10, t(7; 11)] | 1 | Shiba et al., 2013 |
| L359V | Somatic | Normal GATA2 expression  Increased transcriptional activation of CD34  Increased binding affinity to the coactivator CBP  Enhanced binding to PU.1  Inhibited the ability of PU.1 to activate CSF1R transcription  Enhanced inhibition of the c-JUN and PU.1 interaction  Inhibited myelomonocytic differentiation and proliferation^6^ | CML with blast crisis, CML with blast crisis (+11p), CML with blast crisis (-17q) | 8 (8 pedigrees) | Zhang et al., 2008 |
| W360L | Somatic | Altered ZF2 domain | CML with blast crisis (ANO5, MAX, ENO1, COL3A1, AFP, SERPINA1, MGAT5B, ZNF208) | 1 | Huang et al., 2014 |
| R361delRNAN | Germline^+^ | In-frame deletion  Partially deleted ZF2 domain | MDS (-7, +8, ASXL1), MonoMAC | 1 | Hsu et al., 2011  West et al., 2014 |
| R361H | Somatic | Altered ZF2 domain | Sporadic AML (NPM1) | 1 | Fasan et al., 2013 |
| R361C | Germline^+^  Germline^+^ | Reduced GATA2 expression  Reduced IKBKG, CXCL12, SRC, NOTCH1 & FERMT3 expression  Increased RUNX1, FYN & ETS1 expression^3^ | DCML, MDS, lymphedema  MDS, MonoMAC | 1  1 | Dickinson et al., 2013  Hsu et al., 2013 |
| R361L | Germline^+^ | Reduced GATA2 transactivation of CD34^3^ | Emberger, developmental delay | 1 | Ostergaard et al., 2011 |
| R362Q | Somatic  Somatic^*^  Somatic  Somatic  Somatic^*^ | Altered ZF2 domain | AML (biCEBPA)  AML (biCEBPA)  AML (mCEBPA, WT1)  AML (WT1, FLT3, ATP2A2, LPL, PRICKLE3, SORCS3)  AML (biCEBPA, WT1, NRAS) | 3 (3 pedigrees)  1  1  2 (2 pedigrees)  1 | Fasan et al., 2013  Green et al., 2013  Shiba et al., 2013  Yan et al., 2011  Luesink et al., 2012 |
| R362P | Somatic  Somatic^*^ | Altered ZF2 domain | AML (KRAS), AML [t(11;19)]  AML (NPM1, NRAS) | 2 (2 pedigrees)  1 | Shiba et al., 2013  Luesink et al., 2012 |
| R362G | Somatic^*^ | Altered ZF2 domain | AML [+8, t(15;17), KIT]  AML (NPM1, NRAS) | 1  1 | Shiba et al., 2013  Luesink et al., 2012 |
| R362fs | Somatic^*^ | Partially deleted ZF2 domain | Paediatric AML (NRAS, -7) | 1 | Luesink et al., 2012 |
| R362dup | Somatic^*^ | In-frame insertion  Altered ZF2 domain | Sporadic AML (mCEBPA) | 1 | Green et al., 2013 |
| D367fs | Germline | Reduced GATA2 expression  Reduced IKBKG, CXCL12, SRC, NOTCH1 & FERMT3 expression  Increased RUNX1, FYN & ETS1 expression^3^ | MDS (ASXL1), MonoMAC | 2 (1 pedigree) | West et al., 2014 |
| N371K | Germline^+^ | Altered ZF2 domain | MonoMAC, MDS (-7, ASXL1) | 1 | West et al., 2014 |
| A372T | Germline^+^  Germline^+^ | Reduced GATA2 expression  Altered ZF2 domain^3^ | DCML  Neutropenia, MonoMAC, AML [(-7, t(11;19)] | 2 (2 pedigrees)  1 | Dickinson et al., 2013  Pasquet et al., 2013 |
| C373R | Germline^+^ | Reduced GATA2 transactivation of CD34^3^ | Emberger, MDS (-7) | 1 | Ostergaard et al., 2011 |
| C373del5 | Germline | In-frame deletion  partially deleted ZF2 domain | MDS, MDS (-7) | 3 (1 pedigree) | Spinner et al., 2013 |
| L375I | Somatic | Altered ZF2 domain | AML [mCEBPA, t(15;17)] | 1 | Shiba et al., 2013 |
| L375F | Germline | Altered ZF2 domain | MonoMAC | 2 (1 pedigree) | West ES et al., 2013 |
| L379Q | Somatic | Altered ZF2 domain | AML (biCEBPA) | 1 | Fasan et al., 2013 |
| P385L | Somatic^*^ | Potential CEBPA binding site | AML (mCEBPA) | 1 | Green et al., 2013 |
| M388V | Germline | Potential sumoylation site | Neutropenia, MonoMAC, MDS (+8, -7q) | 2 (1 pedigree) | Pasquet et al., 2013 |
| M388T | Germline | Reduced GATA2 expression  Reduced IKBKG, CXCL12, SRC, NOTCH1 & FERMT3 expression  Increased RUNX1, FYN & ETS1 expression.  Potential sumoylation site^3^ | MonoMAC, severe infections | 3 (1 pedigree) | Hsu et al., 2013 |
| 390delK | Unknown | In-frame deletion  Acetylation site  Potential sumoylation site | DCML, MDS (biGATA2 with G200fs mutation) | 1 | Dickinson et al., 2013 |
| R396Q | Germline  Germline^+^  Germline  Germline | Reduced GATA2 expression  Reduced IKBKG, CXCL12, SRC, NOTCH1 & FERMT3 expression  Increased RUNX1, FYN & ETS1 expression  Increased WT1 expression^3^ | MDS, AML  Emberger/MonoMAC  Neutropenia, MonoMAC, MDS (-7), AML (+11, -7)  MDS (+8), MDS, MonoMAC, severe infections | 3 (1 pedigree)  1  4 (1 pedigree)  8 (4 pedigrees) | Holme et al., 2012  Ishida et al., 2012  Pasquet et al., 2013  Spinner et al., 2013 |
| R396W | Germline^+^ | Reduced GATA2 expression  Reduced IKBKG, CXCL12, SRC, NOTCH1 & FERMT3 expression  Increased RUNX1, FYN & ETS1 expression^3^ | MonoMAC, MDS (+8), MDS | 2 (2 pedigrees) | Spinner et al., 2013 |
| R398Q | Germline | Altered ZF2 domain | DCML, MDS (-7) | 6 ( 1 pedigree) | Dickinson et al., 2013 |
| R398W | Germline  Germline | Reduced GATA2 expression May prevent a critical interaction with the minor groove of DNA.  Reduced IKBKG, CXCL12, SRC, NOTCH1 & FERMT3 expression  Increased RUNX1, FYN & ETS1 expression^3^ | DCML, MDS  MDS, CMML (ASXL1), MDS (+8), LGL, MonoMAC | 5 (3 pedigrees)  6 (5 pedigrees) | Dickinson et al., 2013  West et al., 2014 |
| Unknown | Germline^+^ | Null allele^4^  Reduced IKBKG, CXCL12, SRC, NOTCH1 & FERMT3 expression  Increased RUNX1, FYN & ETS1 expression | MDS, LGL, MonoMAC, MDS/AML (+8) | 3 (3 pedigrees) | Hsu et al., 2013 |
